# Evaluation of the community involvement of nursing experts in reducing unintentional injuries in children

**DOI:** 10.1186/s12887-022-03700-9

**Published:** 2023-01-24

**Authors:** Li-Fang Qian, Ting-Ting Cheng, Hong-Xia Chen, Dong-Hui He, Xiao-Min Peng, Qing-Hua Zhao

**Affiliations:** Department of Nursing, People’s Hospital of Chizhou, Chizhou, 247000 China

**Keywords:** Infant, Unintentional injury, School for pregnant women, Vaccination

## Abstract

**Background:**

Nursing experts regularly visited the community to deliver safety education on the prevention of unintentional injuries in children to the parents of children aged 0–6 years and to pregnant women in a maternity school. This was undertaken to explore the effects of the measure on preventing unintentional injuries in children in Chizhou, China.

**Methods:**

Using the convenience sampling method, the guardians(it means mother in this study)of children were investigated. The nursing experts visited communities in which the number of nursing experts is declining. Data on unintentional injuries in children in the previous year were collected retrospectively.

**Results:**

After the nursing experts delivered safety education to the community, the scores of the questionnaire on unintentional injury prevention knowledge completed by children’s guardians increased significantly (*p* < 0.01). Among the children whose guardians completed the questionnaire, there were 157 cases of unintentional injury in 2020 and 103 cases in 2021 (*p* < 0.05). The types of unintentional injuries included scratches, falls, sharp object injuries, swallowing of foreign bodies, burns and traffic accidents; there was no statistical difference (*p* > 0.05). However, there were significant differences in terms of gender ratio and location (*p* < 0.05).

**Conclusion:**

In conjunction with the maternity school for pregnant women and the vaccination programme, nursing experts delivered safety education regarding unintentional injuries in children; this may have promoted safety and protection awareness in the children’s guardians and reduced unintentional injuries.

## Background

Unintentional injury is a major problem that threatens the health of children in China. Globally, approximately 950,000 children die from unintentional injuries every year [1]. According to the World Health Organization’s *World Report on Child Injury Prevention*, drowning deaths comprise the vast majority of cases (98%) []. Unintentional injury is also the primary risk factor for health problems among children aged 0–6 years in many regions of China [2]. Since the 1970s, community nurses have focused on children’s health by establishing services and applying childcare strategies [3]. Recently, the role of child health nurses has involved the consideration of health promotion together with prevention methods [4]. Furthermore, community nurses have the responsibility to provide home visits and focus on the psychosocial and mental issues of children [5].

According to the standards of the People’s Republic of China, the causes of unintentional injuries in children are traffic accidents, crush injuries, fall injuries, scratches, puncture cuts and other injuries [6]. Severe unintentional injury causes serious physical and mental trauma to children, while their disability and death bring heavy burdens and losses to both families and society [6]. The Chinese government aims to work hard to protect people’s health in a full cycle and transform the treatment of diseases into a health-centred approach. Safety education plays a significant role in reducing the incidence of unintentional injuries in children, but many guardians do not have time to participate in safety education. Therefore, it is meaningful to investigate the delivery of safety education to guardians during their attendance at community hospitals for childhood vaccinations. This study explores the feasibility of delivering safety education in community hospitals by evaluating the preventive effect of safety education carried out by community nursing experts on accidental injuries.

## Methods

### Research subjects

This study was conducted at the People's Hospital of Chizhou between January 2020 and January 2021. The subjects included in the January 2020 questionnaire survey were the guardians of children aged 0–6 years. The inclusion criteria were as follows: 1) those who participated voluntarily, 2) those who were in good health and 3) those with a high school education or above. The exclusion criteria were 1) those with a mental illness and 2) those who were unable to communicate effectively. Based on the above criteria, the target added a special trainer for infant safety education, which was delivered by nursing experts in the declining community during the vaccination period. This study was approved by the ethics committee of the People's Hospital of Chizhou(Ethics approval number: 2020-KY-07), and all participants provided signed informed consent.

## Methods

### Sampling method

The method of convenience sampling was used in this study. The survey community was the community connected to the nursing experts from our hospital. In January 2020 and January 2021, special personnel surveyed the guardians of children who attended for vaccinations in the community. The questionnaire was self-completed by the guardians, and a total of 880 questionnaires were distributed. A total of 741 valid questionnaires were returned, with an effective response rate of 84.20%. Based on the results of the valid questionnaires, a dedicated person retrospectively collected data on the unintentional injuries in children in the community over the previous year.

### Educational method

In 2020, per the deployment of the Municipal Health Commission, our hospital arranged for two nursing experts (specialising in women and children) to venture into the community. To effectively prevent unintentional injuries in children, the two nursing experts provided health education for expectant mothers of at least 32 weeks gestation in the community maternity school. The parents of children aged 0–6 years were provided with education on unintentional injury safety in instalments, which were completed on time.

We consulted a wide range of literature related to children's accidental injuries, screened out children's accidental injuries with high frequency, communicated with the expert team and finally determined the content of the training lecture. The lecture covered the types of common accidental injuries to children, including those occurring in different environments (home, school, amusement park, park, etc.) and how to prevent them. The lectures were conducted in the form of on-site talks and interactions. Each lecture lasted for 1 h.

For the pregnant women in the maternity school, their education time was every Wednesday morning. At the same time, pregnant women with a gestational age of more than 32 weeks were screened. The nursing experts gave safety education lectures at a vaccination observation point every Tuesday and Friday morning to the guardians of children who were being vaccinated. The education and training paid special attention to typical case education, such as social reports and unintentional injury cases of children treated by the hospital. On-site interactive simulations provided corresponding practical experience based on the prevention and treatment of various unintentional injuries.

### Investigation method

Using descriptive research methods, the self-designed ‘Questionnaire on the Status Quo of unintentional injury Knowledge of children within One Year’ was used for the investigation. The questionnaire was designed by nursing experts and safety trainers, and the content was divided into three parts [7]: (1) basic information about children at home, including birth time, name, mother’s age, mother’s education level and the main caregivers, (2) unintentional injuries in children within 1 year, including the cause, location and basic treatment measures, (3) unintentional injury knowledge questionnaire with infant knowledge points (total of 100 points), including the causes, preventive measures and basic handling of unintentional injuries in children. There were 10 items in total; each item was divided into 5 levels, with 2 points for each level.

In January 2020, a questionnaire survey of 356 subjects who met the requirements for community vaccinations was conducted. In January 2020, a questionnaire survey was conducted on 385 eligible research subjects. After the investigation, a person was assigned to register the age of the children, their gender, the cause and location of their injuries and other data. Multiple unintentional injuries to an infant were counted as one person for data entry purposes.

### Statistical analysis

All the original data were entered into a database established using EpiData 3.0. The data were processed by SPSS 19.0 statistical software. The measurement data were compared by a test between groups. The count data were compared by a rank–sum test, and the comparison between groups was completed by a Chi-squared analysis. A value of *p* < 0.05 indicated that the difference was statistically significant. The Cronbach α coefficient method was used to measure the internal consistency reliability. An α value of > 0.8 indicated excellent internal consistency, 0.6 ~ 0.8 indicated good consistency, and a value of < 0.6 indicated poor internal consistency. The validity analysis mainly examined the content and structural validity and identified structural validity via a multiple factor analysis (MFA). The questionnaire consisted of 5 parts, each of which involved 15–32 problems, which could be selected from multiple-choice answers. Therefore, the questionnaire used the MFA statistical method.

## Results

### Reliability and validity

The α coefficient was 0.826, which indicated good reliability. The questions in the questionnaire were on common accidental injuries received at home, school and in outdoor activities; how to prevent accidental injuries and how to deal with accidental injuries. There were five dimensions. The cumulative contributions of the four principal components extracted in the five dimensions were 91.24%, 91.16%, 92.42%, 90.61% and 91.55%. The results of the factor analysis showed that the scale had a clear structure and good structural validity.

### Infant guardians’ unintentional injury knowledge questionnaire scores

The results are shown in Table 1. The score of the questionnaire survey on unintentional injury prevention knowledge completed by the children’s guardians after education was 95.79 ± 4.34, which was higher than before the announcement (86.47 ± 1.81). The comparison was statistically significant (*p* < 0.05).

**Table 1:** Infant guardian accidental injury knowledge questionnaire scores (points)

Group	Cases	Score
before safety education	356	86.47 ± 1.81
after safety education	385	95.79 ± 4.34
*P* value		< 0.05

### Comparison of unintentional injuries among children in the community

The results are presented in Table 2. The incidence of unintentional injuries among children in 2021 was 26.75%, which was lower than in 2020 (44.10%) (*p* < 0.05).

**Table 2:** Accidental injuries of infants in a community before and after safety education (cases, %)

Group	Cases	Accidental injury occurred	No accidental injury occurred
before safety education	356	157(44.10)	199(55.89)
after safety education	385	103(26.75)	282(73.25)
*P* value		< 0.05	

### Analysis of the causes of unintentional injuries to children

The results are shown in Table 3. The causes of unintentional injuries included abrasions, falling injuries, sharp force injuries, burns and scalds, ingestion of foreign bodies and traffic accidents. After the community safety education was delivered, the incidence of falling injuries, ingestion of foreign bodies, burns, scalds, scratches, sharp instrument injuries and traffic accidents decreased. There was no statistical difference (*p* > 0.05).

**Table 3:** Analysis of the causes of accidental injuries in infants (cases, %)

Causes of accidental injuries	Before safety education		After safety education		*P* value
	cases	%	cases	%	
scratches	47	12.21	39	10.13	> 0.05
fall injuries	32	8.31	14	3.64	
sharp weapon injuries	28	7.27	20	5.19	
burns	20	5.19	10	2.60	
foreign body swallowing	18	4.68	9	2.34	
traffic accidents	12	3.12	11	2.86	

### Gender and location of children with unintentional injuries

The results are presented in Table 4. The ratio of unintentional injuries among boys was higher than in girls. The ratio of boys in 2021 was lower than in 2020, but there was no significant difference (*p* > 0.05). The rate of infant unintentional injuries in the family decreased from 52.87% in 2020 to 35.92% in 2021, which was statistically different (*p* < 0.05).

**Table 4:** Sex and location of accidental injury infants (cases, %)

Group	Cases	Gender		Location	
		male	female	at home	outside home
before safety education	157	103(65.61)	54(34.39)	83 (52.87)	74 (47.13)
after safety education	103	55(53.40)	48(46.60)	37 (35.92)	66 (64.08)
*P* value		> 0.05		< 0.05	

## Discussion

To effectively evaluate children’s health conditions and prevent childhood injuries, community assessment and intervention by community nurses are required as a unit of care. It has been reported that community health nurses provide help to all families with children in Australia [8]. These nurses, who have postgraduate qualifications, possess the ability to give support for free to the health of children. Furthermore, since they also possess solid professional knowledge on how to take care of newborns and young children, they can provide extensive professional advice to families [9]. In addition, these community nurses visit families, check the health condition of the children, provide health education and offer telephone consultations [10]. A previous study suggested that the nutritional status of children in Africa could be assessed promptly and accurately by community-based nursing [11].

National policies have begun to intervene in the prevention of unintentional injuries [12]. In the past 2 years, the Ministry of Education had launched a safety education platform for schools, focusing on students’ self-participation. The incidence of unintentional injuries among children in our hospital was relatively high; children accounted for 43%, which was consistent with Zhao Jingmei’s report [13]. Therefore, our hospital organised the collaboration of nursing experts from relevant departments to tackle the city’s situation and deliver safety education in urban communities. The hospital expanded its audience for the education programme to include pregnant women with a gestational age of more than 32 weeks as well as guardians of children being vaccinated. Pregnant women with a gestational age of more than 32 weeks are close to giving birth, and for these women, the safety and care of children are what they urgently need.

The guardians of children being vaccinated needed to observe their infants for half an hour after the vaccinations, and they were given infant safety education. In their presentations, nursing experts in the declining community focused on case education and on-site simulations. On-site guardians participated together and simultaneously completed the questionnaire surveys. In this study, the safety presentations in declining communities corresponded to the audience’s understanding of the knowledge. Consequently, the questionnaire score improved significantly. Furthermore, the community’s implementation of this safety education played a positive role in strengthening the prevention of unintentional injuries by parents.

The top five unintentional injuries in children were from falls, sharp objects, burns and scalds, swallowing of foreign bodies and traffic accidents. These unintentional injuries mainly occurred at home before the safety education was delivered. Before the education, the safety awareness of caregivers was not in place. A lack of safety knowledge was the main reason for unintentional injury, and the safety of the children depended entirely on the parents or caregivers [14]. However, even if parents or guardians might have thought it necessary to let go and don’t look at the baby, they should be able to foresee dangers in the environment around them and pay attention to possible hazards from food and household products. Therefore, the protective measures of the content meant for the declining community mainly stated that the guardians of children should strengthen the prevention of unintentional injury, especially in the home environment. Hence, people would find it easier to control and improve the prevention of fall injuries, the swallowing of foreign bodies and the occurrence of burns. For example, people could fit guardrails in places where children might find it easy to climb and fall, properly store small objects to prevent them from being swallowed and improve the temperature management of food and drink at home to prevent children from being scalded. Through safety education, the guardians gained a better sense of safety only after they had fully understood the potential dangers.

Anticipating that something may be dangerous creates a defensive response to the potential danger, for example, not locking young children indoors when they are alone and adding guardrails to balconies. Many parents of children pay much less attention to children’s safety education than their intellectual development [10], and these details are often ignored by young parents.

In this study, parents and caregivers were given detailed safety education. People confronted the potential safety risks faced by children at different stages of activity and the emergency treatment measures for various safety accidents, which effectively improved the awareness of parents and caregivers in preventing accidents, minimised the occurrence of unintentional injury and reduced the harm caused by unintentional injury.

Children’s unintentional injuries were more common in boys. This result was similar to the findings of Wang Wenchao's research. The physical and psychological development of young children is rapid, especially after the age of 3 years, when boys are naughty, active, courageous, adventurous and sporty. As children get older, it becomes more difficult for guardians to protect them when they participate in activities outside of the home. Children cannot recognise dangers as their parents or guardians can; therefore, boys are relatively more injured in accidents than girls. From Table 4, it can be seen that community activities as guardians still need to implement safety measures and interventions for children of different sex.

Finally, this study did not include a control group which should be included in future research.

## Conclusions

In combination with a maternity school for pregnant women and a vaccination programme, nursing experts delivered safety education regarding unintentional injuries in children, which might have improved the safety and protection awareness of children’s guardians and reduced unintentional injuries. In the future, people can continue to use community resources to establish consortia, and they can promote public health information via publicity modes. Additionally, they can raise awareness of the potential risks of unintentional injury and strengthen knowledge by providing education on unintentional injury prevention [15].

## Data Availability

The datasets used and analyzed during the current study are avaliable from the corresponding author on reasonable request.
